# Characterization of *Streptomyces* Cell Surface by the Microbial Adhesion to Solvents Method

**DOI:** 10.1155/2023/8841509

**Published:** 2023-05-11

**Authors:** C. Zanane, S. Mitro, D. Mazigh, S. Lekchiri, T. Hakim, M. El Louali, H. Latrache, H. Zahir

**Affiliations:** Industrial and Surface Engineering, Research Team of Bioprocesses and Biointerfaces, Faculty of Sciences and Techniques, Sultan Moulay Slimane University, Beni Mellal, Morocco

## Abstract

The cell surface physicochemical properties of *Streptomyces* should influencing the dispersal and adsorption of spores and hyphae in soil and should conditioning there interactions with organic or metal substances in the bioremediation of contaminated environment. These properties are concerning surface hydrophobicity, electron donor/acceptor, and charge surface. To date, only hydrophobicity of *Streptomyces* was studied by contact angle measurements and microbial adhesion to hydrocarbons (MATH). In this work, we studied the electron donor/acceptor character of the *Streptomyces* cell surface in two ionic strength 10^−3^ M and 10^−1^ M of KNO_3_. Thus, to facilitate the characterisation of the surfaces of microbial cells, we used a simple, rapid, and quantitative technique, the microbial adhesion method to solvents (MATS), which is based on the comparison of the affinity of microbial cells for a monopolar solvent with a polar solvent. The monopolar solvent can be acid (electron acceptor) or basic (electron donor), but both solvents should have a surface tension similar to that of the Kifshitz van der Waals components. At the significant ionic strength of the biological medium, the electron donor character is well expressed for all 14 *Streptomyces* strains with very significant differences among them ranging from 0% to 72.92%. When the cells were placed in a solution with a higher ionic strength, we were able to classify the donor character results into three categories. The first category is that the weak donor character of strains A53 and A58 became more expressed at 10^−1^ M KNO_3_ concentration. The second category is that three strains A30, A60, and A63 expressed a weaker character in a higher ionic strength. For the other strains, no expression of the donor trait was obtained at higher ionic strength. In a suspension with a concentration of 10^−3^ KNO_3_, only two strains expressed an electron acceptor character. This character is very important for strains A49, A57, A58, A60, A63, and A65 at 10^−1^M KNO_3_. This work has shown that these properties vary greatly depending on the *Streptomyces* strain. It is important to consider the change in physicochemical properties of surface cells with ionic strength when using *Streptomyces* in different bioprocesses.

## 1. Introduction

*Streptomyces* are Gram-positive filamentous bacteria belonging to the phylum Actinobacteria [1]. They are ubiquitous in a variety of natural and artificial environments and constitute a large fraction of soil microbial populations [2, 3]. These bacteria are characterized by a complex and peculiar developmental cycle [1, 4]. *Streptomycetes* are extensively studied as producers of a wide variety of natural metabolites of biotechnological interest [5–8]. They produce about 75% of commercially and medically useful antibiotics and about 60% of those developed for agriculture [9, 10]. However, few studies have highlighted the interfacial interactions between this bacterium and its environment that could play a critical role in the process of producing bioactive molecules and bioenvironment activities [11, 12]. Van der Waals electrostatic and acid-base interactions are associated with bacterial adhesion phenomena depending on the physicochemical properties of the substrate and the bacterial surface such as hydrophobicity and electron donor/acceptor properties [13–17].

Two methods the contact angle method and the microbial hydrocarbon adhesion method (MATH) were used to determine the hydrophobicity of the bacterial surface of *Streptomyces*. Compared to MATH, the contact angle method, which consists in estimating the surface energy by measuring the contact angle of the drops deposited on the studied surfaces, requires very elaborate, specific equipment [18–20]. In the case of *Streptomyces*, the microbial hydrocarbon adhesion method (MATH) was first used by and more recently by Ding and Lammler [21] and N. Alonso [22] to evaluate the surface hydrophobicity.

However, van Oss [23] and Bellon Fontaine et al. [13] explained that acid-base interactions are expected to play a decisive role in the interactions between two materials and their importance in polar media and is very important especially in aqueous media.

The contact angle method with different solvents is commonly used to estimate the electron donor/acceptor [24] characteristics of the partially dehydrated cell surface, but the method proposed by Bellon Fontaine et al. [13], called microbial solvent adhesion (MATS), has proven to be a simple, rapid, and quantitative method and may be advantageous as long as the cells are fully hydrated in an aqueous medium.

The MATS method is based on the comparison of the affinity of microbial cells to monopolar and apolar solvents used to determine the electron donating (basic) and accepting (acidic) properties of microbial cells. Van Oss [25] reported that the acid-base interactions are 10 to 100 times more important than the other interactions. Despite the crucial importance of these properties, there are no noticeable discussions about the methods that could be used for its actual evaluation.

Few works have studied the physicochemical parameters of the cell surfaces of *Streptomyces*. Thus, to our knowledge, no study by MATS has been used to characterize donor/acceptor of *Streptomyces*. The objective of this work is to determine for the first time the electron donor/acceptor character by MATS (microbial adhesion to solvents) to complement the solvent adhesion properties of 14 strains of *Streptomyces* under two ionic strengths, in order to extend the knowledge on this important bacterial genus and to have a better evaluation and control of the adhesion mechanisms and the biofilm formation in biotechnology. Considering different bioprocess sectors and different natural or industrial environments of *Streptomyces*, two ionic strengths were tested for their influence on physicochemical surface properties.

## 2. Materials and Methods

### 2.1. Bacterial Strains, Growth Conditions

The fourteen strains of *Streptomyces* sp used in this study were isolated from soils of the Beni Amir region of Morocco [26, 27]. These strains were grown on BENNET medium (10 g D-glucose, 1 g yeast extract, 1 g meat extract, 2 g peptone, 15 g agar, and 1 litre distilled water, and pH of the medium is about 7.5) at 28°C for three weeks. For the rich culture, approximately 100 ml of liquid BENNET medium for each strain is incubated under agitation for 7 days in an incubator at 28°C.

### 2.2. Preparation of Bacterial Suspension

After incubation, the cultures were subjected to two successive centrifugations (8600 g for 15 min), to remove the culture medium and wash the cells. The pellets were resuspended in KNO_3_ solution at two different ionic strengths of 10^−3^ or 10^−1^ M. Then, the suspension was subjected to Stomaquar for 20 minutes to disperse the mass of *Streptomyces* and avoid their aggregation. The concentration of each bacterial suspension was adjusted by measuring the optical density (OD) at 405 nm between 0.7 and 0.8 corresponding to 10^8^ CFU/ml with an ELISA spectrophotometer (Multiskan EX, Labsystems).

### 2.3. Microbial Adhesion to Solvents

In 1996, Bellon-Fontaine et al. [13] were expanded this method MATS to estimate donor and acceptor electron.

According to Bellon-Fontaine et al. [13], the MATS method is based on the comparison of the affinity of microbial cells for a monopolar solvent and an apolar solvent and is used to determine the electron donating (basic) and electron accepting (acidic) properties of microbial cells.

Experimentally, the bacteria were suspended at 0.7 to 0.8 to 405 nm optical density (approximately 10^8^ CFU/m1 cell density) in KNO_3_ 10^−3^M or in 10^−1^M. 2.4 ml of each bacterial suspension was vortexed for 90 s with 0.4 ml of the solvent. The mixture was allowed to stand for 15 minutes to ensure complete separation of the two phases. The optical density of the aqueous phase was later measured by spectrophotometer.

The percentage of bound cells (i.e., adhesion percentage) to each solvent was then calculated using the following formula: % adhesion = (1 − (*A*/*A*_0_)) × 100 where *A*_0_ corresponds to the absorbance measured at 405 nm for the bacteria suspension before mixing and *A* to the absorbance after mixing.

The pairs of solvents, as described by Bellon-Fontaine et al. [13], were used: chloroform, an electron acceptor solvent, and hexadecane, a nonpolar solvent; and diethyl ether, a strong electron donor solvent, and hexane, a nonpolar solvent. Due to the similar Lifshitz–van der Waals components of the surface tension in each pair of solvents, differences among the results obtained with chloroform and hexadecane, on one hand, and between diethyl ether and hexane, on the other hand, would indicate the electron donor and electron acceptor character of the bacterial surface, respectively. The percentage of cells adhered to hexadecane was used as a measure of cell surface hydrophobicity.

### 2.4. Statistical Analysis

Data were statistically analyzed using one-way analysis of variance (ANOVA), by SPSS (Statistical Program for Social sciences) version 20.0 for Windows. All analyses were performed in triplicate (*n* = 3), and data were presented as means ± standard deviation (SD), comparing mean values of strains.

## 3. Results

In this work, we studied the physicochemical properties of the cell surfaces of 14 strains of *Streptomyces* in order to contribute to a better understanding of the interactions between these bacteria and their environment. Therefore, the determination of acid-base properties (electron donor and acceptor) could be of major importance in the phenomenon of microbial adherence [23]. The theory of Van Oss et al. [28] thus considers that Lifshitz–van der Waals interactions, electrostatic interactions, and Lewis acid-base interactions are the three fundamental interactions involved in the phenomena of adhesion of micro-organisms to support. These properties are determined for the first time using the MATS method. In this method, the affinity of bacteria for polar solvents is considered to be the result of the conjunction of electrostatic interactions, Van der Waals interactions, and electron donor/acceptor interactions. Thus, in the absence of electrostatic interactions, differences in microbial adhesion to apolar and polar solvents may be matched to favorable or unfavorable acid-base interactions. Favorable interactions are indicated by an increase in bacterial affinity for a monopolar solvent and vice versa [13].

### 3.1. Hydrophobic Character by Adhesion to Hexadecane

According to the literature, hexadecane is considered to be a highly pure apolar solvent [13], which allows an efficient separation [29]. Thus, hexadecane is mostly used because of the great affinity of bacterial cells to it [30].

**Figure 1:**
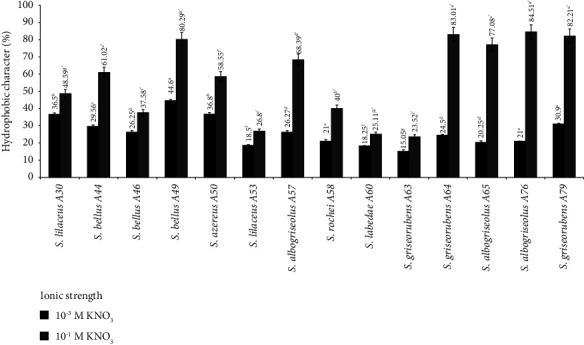
Hydrophobic character of the 14 strains of *Streptomyces* to hexadecane at two ionic strengths: 10^−3^ M and 10^−1^ M of KNO_3._ The letters a, b, c, d, e, f, g used to indicate statistical groups at ionic strength 10^−3^ M of KNO_3_, and the letters “a,” “b,” “c,” “d,” “e,” “f,” “g,” “h,” “i,” “j,” “k,” “l” used to indicate statistical groups at ionic strength 10^−1^ M of KNO_3_. Data were presented as means ± standard deviation (SD).

The percentage of *Streptomyces* cells to hexadecane is presented in Figure 1.

At the ionic strength of 10^−3^M KNO_3_, all *Streptomyces* strains show a low affinity for hexadecane, which shows a relatively hydrophilic character. On the other hand, at the high ionic strength of 10^−1^M KNO_3_, the affinity to hexadecane becomes more expressed, which proves the hydrophobic character of the studied strains but for A30, A46, A53, A58, A60, and A63, a low increase was observed (Figure 1).

### 3.2. Electron Donor/Acceptor Character of the Surfaces of Streptomycetes

The electron donor/acceptor properties were estimated by comparing the affinity of the cells to monopolar and apolar solvents that have similar Lifshitz–van der Waals surface tension components. At low ionic strength, the values of the Lewis acid-base properties estimated by the MATS method do not only reflect the electron donor or acceptor properties of the cellular surface but also electrostatic forces.

**Figure 2:**
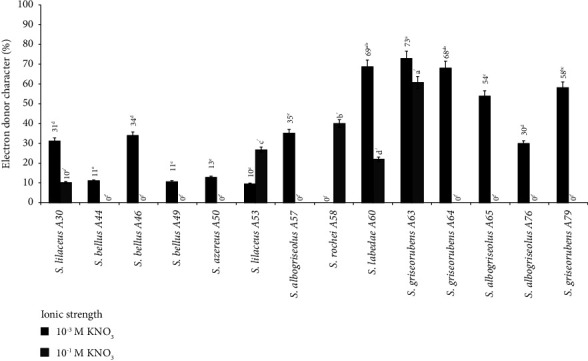
Electron donor character of the 14 strains of *Streptomyces* at two ionic strengths: 10^−3^ M and 10^−1^ M of KNO_3._ The letters a, b, c, d, e, f used to indicate statistical groups at ionic strength 10^−3^ M of KNO_3_, and the letters “a,” “b,” “c,” “d,” “e,” “f” used to indicate statistical groups at ionic strength 10^−1^M of KNO_3_. Data were presented as means ± standard deviation (SD).

The difference between the percentage of adhesion to chloroform and the percentage of adhesion to hexadecane allows the estimation of the electron donor character (basicity). The cell surface is only considered an electron donor when this difference is positive [31]. The results of the electron donor character obtained for *Streptomyces* strains under different ionic strength are presented in Figure 2.

At the low ionic strength of 10^−3^ M KNO_3_ at pH 7, all *Streptomyces* strains showed a maximum affinity for the chloroform solvent compared to the apolar solvent. The difference between the percentage of adherent cells for the polar (chloroform) and apolar (hexadecane) solvent is always positive, with the exception of strain A58, which shows a null character. At the high ionic strength 10^−1^ M of KNO_3_, we observe that the affinity to chloroform is higher than to hexadecane. These results show an electron donor character expressed for strains A30, A53, A58, A60, and A63 (Table 1).

**Figure 3:**
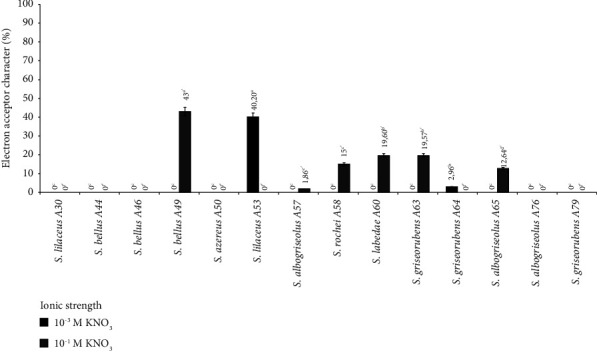
Electron acceptor of the 14 strains of *Streptomyces* to hexadecane at two ionic strengths: 10^−3^ M and 10^−1^ M of KNO_3._ The letters a, b, c used to indicate statistical groups at ionic strength 10^−3^ M of KNO_3_, and the letters “a,” “b,” “c,” “d,” “e,” “f” used to indicate statistical groups at ionic strength 10^−1^ M of KNO_3_. Data were presented as means ± standard deviation (SD).

The electron acceptor character is estimated by the difference between the affinity of the microbial cell for diethyl ether and for hexane. Only positive values are considered to have an electron acceptor character [13]. The results of electron acceptor character obtained for *Streptomyces* strains under different ionic strengths are presented in Figure 3.

For the 10^−3^M ionic strength of KNO_3_ at pH 7, with the exception of strains A53 and A64, the affinity of all strains to the apolar solvent (hexane) is higher than to the polar solvent diethyl ether, indicating a null acceptor character. For the 10^−1^ M ionic strength, the affinity for diethyl ether is higher than for hexane for strains A49, A58, A60, A63 and A65, which shows that they have a relatively strong acceptor character, whereas for the other strains, this character is null (Table 2).

## 4. Discussion

The hydrophobicity of the microbial cell surface is an important factor in the adhesion phenomenon [32, 33]. The hydrophobic/hydrophilic character is determined by the percentage of cells attached to hexadecane. The surface is considered relatively hydrophobic when this percentage is higher than 50% and relatively hydrophilic in the opposite case [13].

Our results are consistent with data published by Hamadi et al. [31] and Hamadi and Latrache [34] which reported that the hexadecane adherence of *Staphylococcus aureus*, *Pseudomonas aeruginosa*, and *Escherichia coli* increases by increasing the ionic strength of KNO_3_ from 10^−3^ M to 10^−1^ M using MATH. However, MATH has been shown to be sensitive to electrostatic and hydrophobic interactions [35, 36]. In general, at neutral pH, all hydrocarbons used in MATH methods give a negatively charged interface with water and most buffers [36, 37].

Nevertheless, the increase in ionic strength induces a decrease in electrostatic charge [38, 39]. This decrease is attributed to the significant adsorption of cations, which could cause the neutralization of charged groups present on the surface. Thus, the overall negative charge of the bacterial surface could promote repulsion of the negatively charged solvent [14]. This can be explained by the Debye length which can also be used to quantify the influence of ions in the solution on the interaction between the bacterial membrane and the hydrophobic surface. If the concentration of ions in the solution is high, the Debye length will be smaller that may decrease the effect of electrostatic interactions between the bacterial membrane and the hydrophobic surface. Indeed, strains that express maximal hydrophobic interactions with hexadecane have a low negative charge on the surface [40]. This could explain the dominance of the hydrophilic character at 10^−3^ M while the hydrophobic character is observed at 10^−1^ M.

Using different methods, it has been reported that generally the degree of hydrophobicity is related to the chemical composition of the surface, the molecular composition of the surface, and their external structures [34, 41–43]. The bacterial membrane is a structure composed mainly of lipids, which have a hydrophilic and a hydrophobic part. When a bacterium is exposed to a hydrophobic surface, the hydrophobic parts of the bacterial membrane can interact with the surface, leading to changes in the structure and function of the membrane.

Hamadi et al. [44] showed, by the contact angle method, that hydrophobicity increases with the level of membrane proteins present on the bacterial surface and decreases with the presence of polysaccharides. In this sense, the work of Claessen et al. [45] and Elliot et al. [46] showed that aerial hyphae and spores of *Streptomyces* are composed of specific surface proteins that allow cells to rise from the liquid medium. These proteins are assembled in such a way that the hydrophobic part is oriented towards the outside. Thus, our study is based on the estimation of the degree of hydrophobicity of *Streptomyces* strains by the MATH method, measuring the percentage of adhesion to hexadecane. Thus, from the chemical composition point of view, we can suggest that the high protein content in these bacteria could be at the origin of their attraction to hexadecane (57% of *Streptomyces* studied).

The acid-base properties are related to the chemical and molecular composition of the cell surface [13, 14]. No work has demonstrated the link between the acid-base properties and the chemical composition of *Streptomyces* cells. Hamadi et al. [31] showed that the difference between the donor character of *Escherichia coli* can be attributed to the presence of basic groups exposed on the cell surface of each bacterium such as carboxyl (COO^−^), lipoproteins and lipopolysaccharides, amines (NH_2_), phosphate (PO_4_) of phospholipids. The work of Hamadi et al. [44] reported that in particular, phosphate groups determined by photoelectron X-ray spectroscopy (XPS) play an important role in determining the electron donor property as measured by the contact angle method, and the electron acceptor characteristic was attributed to the presence of amine groups exposed to the cell surface, such as R-NH or R-OH. These amine groups were responsible for the decrease in the electron (acidic) acceptor property assessed by the same method [47].

Othmany et al. [19] studied the electron donor and acceptor character of six *Streptomyces* strains using the contact angle method. They reported that these strains have a stronger electron donor character and a weak acceptor character. These results are in agreement with those of Maataoui et al. [20] who worked on two *Streptomyces* strains evaluated by contact angle method. In the same direction, we also found that the donor character of eleven *Streptomyces* strains was stronger than the acceptor using the MATS method, except for the strains A49, A57, and A65. These results show that at the high ionic strength of 10^−1^ M KNO_3_, 80% of the *Streptomyces* strains studied show a greater electron donor character than the acceptor character (almost 20%). However, no work has demonstrated the link between the acid-base properties and the chemical composition of *Streptomyces* cells.

The contact angle method combined with the Van Oss equation allows the quantitative determination of acid-base character and hydrophilic/hydrophobic surface properties [48]. Meanwhile, the MATS method gives a qualitative measurement of the electron donor/acceptor properties and is considered to be the result of an inference of the other properties [31]. Moreover, by the MATS method, the cells were suspended in aqueous phase, but by the contact angle method, the cells were dried. This could partly explain the differences in results obtained by the two methods.

Six *Streptomyces* strains A30, A44, A46, A50, A76, and A79 showed zero acceptor character for both ionic strengths. For other Gram-positive bacteria, *Staphylococcus aureus* showed zero electron acceptor character for both 10^−3^ M and 10^−1^ M ionic strengths of KNO_3_ [31, 34] and weak electron donor character for *Listeria monocytogenes* under slightly acidic conditions [14].

## 5. Conclusion

The electron donor/electron acceptor character plays a very important role in the microbial adhesion phenomenon. This work has shown that these properties are very variable depending on the strains which would help to complete the technological power of *Streptomyces* for their ability to bring into play interactions with inert supports. Moreover, and particularly for *Streptomyces* used in bioremediation and in soil, it is very interesting to consider the physicochemistry of the medium such as the ionic strength to modulate these interactions.

## Figures and Tables

**Table 1:** Affinities of *Streptomyces* cells for chloroform and hexadecane used in the MATS analysis under two different ionic strengths: 10^−3^ M and 10^−1^ M of KNO_3_.

Strains	(%) Affinity with solvents			
	Ionic strength 10^−3^ M KNO_3_		Ionic strength 10^−1^ M KNO_3_	
	Chloroform	Hexadecane	Chloroform	Hexadecane
*S. lilaceus* A30	67.30^bc^ ± 0.02	36.51^b^ ± 1.02	58.30^c^ ± 0.01	48.50^g^ ± 0.59
*S. bellus* A44	40.63^f^ ± 2.77	29.56^c^ ± 0.98	0.00^f^ ± 0.00	61.02^e^ ± 0.01
*S. bellus* A46	60.19^cde^ ± 7.44	26.25^d^ ± 1.01	0.00^f^ ± 0.00	37.59^i^ ± 1.52
*S. bellus* A49	55.31^de^ ± 0.01	44.60^a^ ± 0.52	0.00^f^ ± 0.00	80.29^b^ ± 1.00
*S. azereus* A50	49.60^ef^ ± 0.56	36.80^b^ ± 0.92	0.00^f^ ± 0.00	58.56^f^ ± 0.32
*S. lilaceus* A53	28.00^g^ ± 1.00	18.50^f^ ± .44	53.59^d^ ± 0.57	26.80^j^ ± 0.10
*S. albogriseolus* A57	61.45^cd^ ± 2.45	26.27^d^ ± 0.99	0.00^f^ ± 0.00	68.39^d^ ± 0.58
*S. rochei* A58	11.33^h^ ± 0.58	21.00^e^ ± 1.00	62.20^a^ ± 1.00	40.01^h^ ± 0.01
*S. labedae* A60	86.83^a^ ± 0.53	18.25^f^ ± 0.24	26.93^e^ ± 0.99	25.11^jk^ ± 0.57
*S. griseorubens* A63	87.95^a^ ± 0.42	15.05^g^ ± 1.00	60.80^b^ ± 0.01	23.52^l^ ± 0.60
*S. griseorubens* A64	92.53^a^ ± 0.15	24.50^d^ ± 0.52	0.00^f^ ± 0.00	83.01^a^ ± 0.01
*S. albogriseolus* A65	74.17^b^ ± 10.66	20.25^ef^ ± 0.99	0.00^f^ ± 0.00	77.08^c^ ± 1.02
*S. albogriseolus* A76	50.81^def^ ± 1.91	21.00^e^ ± 0.00	0.00^f^ ± 0.00	84.51^a^ ± 0.66
*S. griseorubens* A79	88.97^a^ ± 0.03	30.90^c^ ± 0.06	0.00^f^ ± 0.00	83.21^a^ ± 0.59

Means ± standard deviation. Statistical significance is defined by a value of *p* < 0.05.

**Table 2:** Affinities of the 14 strains of *Streptomyces* for diethyl ether and hexane used in the MATS analysis under two ionic strengths: 10^−3^ M and 10^−1^M of KNO_3_.

Strains	(%) Affinity with solvents			
	Ionic strength 10^−3^ M KNO_3_		Ionic strength 10^−1^ M KNO_3_	
	Diethyl ether	Hexane	Diethyl ether	Hexane
*S. lilaceus* A30	0.00^c^ ± 0.00	26^c^ ± 0.57	0.00^e^ ± 0.00	30^b^ ± 1.73
*S. bellus* A44	0.00^c^ ± 0.00	19.50^f^ ± 0.06	0.00^e^ ± 0.00	22^cd^ ± 1.00
*S. bellus* A46	0.00^c^ ± 0.00	20.25^f^ ± 0.01	0.00^e^ ± 0.00	24.23^c^ ± 0.02
*S. bellus* A49	0.00^c^ ± 0.00	36.08^a^ ± 1.00	42.95^a^ ± 0.99	20.25^d^ ± 0.05
*S. azereus* A50	0.00^c^ ± 0.00	29.60^b^ ± 0.53	0.00^e^ ± 0.00	15.60^e^ ± 0.6
*S. lilaceus* A53	40.20^a^ ± 1.00	18.50^fg^ ± 0.57	0.00^e^ ± 0.00	39.25^a^ ± 0.11
*S. albogriseolus* A57	0.00^c^ ± 0.00	22.40^de^ ± 0.01	18.60^b^ ± 0.59	8^f^ ± 2.00
*S. rochei* A58	0.00^c^ ± 0.00	17.30^gh^ ± 1.73	15^c^ ± 1.00	7.50^f^ ± 0.20
*S. labedae* A60	0.00^c^ ± 0.00	15.74^h^ ± 0.01	19.60^b^ ± 0.59	0^h^ ± 0.00′
*S. griseorubens* A63	0.00^c^ ± 0.00	12.80^i^ ± 0.01	19.57^b^ ± 0.99	0^h^ ± 0.00′
*S. griseorubens* A64	12.96^b^ ± 0.01	9^j^ ± 1.00	0.00^e^ ± 0.00	0^h^ ± 0.00′
*S. albogriseolus* A65	0.00^c^ ± 0.00	16.23^h^ ± 1.02	12.64^d^ ± 0.52	2^gh^ ± 1.52
*S. albogriseolus* A76	0.00^c^ ± 0.00	15.40^h^ ± 0.12	0.00^e^ ± 0.00	0^h^ ± 0.00′
*S. griseorubens* A79	0.00^c^ ± 0.00	22.6^d^ ± 0.56	0.00^e^ ± 0.00	0^h^ ± 0.00′

Means ± standard deviation. Statistical significance is defined by a value of *p* < 0.05.

## Data Availability

The data supporting the current study are available from the corresponding author upon request.
